# Intrinsic and network mechanisms involved in balanced firing and striatal synchrony during dopamine depletion

**DOI:** 10.1186/1471-2202-14-S1-P27

**Published:** 2013-07-08

**Authors:** Sriraman Damodaran, Kim T Blackwell

**Affiliations:** 1 Molecular Neuroscience Department, The Krasnow Institute for Advanced Study, George Mason University, Fairfax, VA, 22030 USA

## 

The input nucleus of the basal ganglia, the striatum, processes cortical inputs in two output streams, called the direct and indirect pathways. Medium Spiny Neurons (MSNs) that express dopamine D1 receptors constitute the direct pathway (D1 MSNs) and MSNs that express dopamine D2 receptors constitute the indirect pathway (D2 MSNs). The two populations of MSNs differ in their intrinsic excitability with D2 MSNs more responsive to somatic current injection [1]. Inhibitory circuits of the striatal network, including feedback inhibition from other MSNs and feedforward inhibition from fast-spiking interneurons (FSIs), modulate this excitability [2,3] to produce balanced firing in response to synaptic input [4]. During dopamine depletion, there is an imbalance in the firing activity and cortico-striatal plasticity between D1 and D2 MSNs, and increased correlation is observed in output structures such as the globus pallidus. Thus, both dopamine and GABA play crucial roles in modulating the activity and properties of these neurons to produce the desired output of the striatum. This study uses computational modeling to investigate the contribution of intrinsic channels and network interactions in modulating the activity and correlation of the striatal network. The network constitutes multi-compartmental models of 500 D1 MSNs, 500 D2 MSNs and 49 FSIs simulated using GENESIS simulation software. The MSNs receive inhibitory input from the other MSNs in the network and from the FSI network. The FSIs are connected to each other by chemical synapses and gap junctions. Similar to experiments, the model D2 MSNs are more excitable than D1 MSNs in response to current injection [1]. Despite this difference, both types of MSNs have similar firing frequencies and latency to spike during up-states in response to synaptic inputs. Removal of either FSI input, or gap junctions between the FSIs, disrupts the balance of firing between D1 and D2 MSNs. In addition, L-type calcium channels are important for balanced firing as blocking these channels or making their conductance equal in D1 and D2 MSNs also disrupts balance of firing. Correlation among MSN is measured in response to a range of cortical input correlation, both in the control and dopamine depleted condition. Simulations reveal that cortical synchrony produces very little increase in striatal synchrony under control conditions, but significantly enhances striatal synchrony in the dopamine depleted condition. This suggests that some of the synchrony in the output structures of the basal ganglia may be caused by enhanced synchrony in the striatum.
